# Protective effect of a nanofilled resin-based coating on wear resistance of glass ionomer cement restorative materials

**DOI:** 10.1186/s12903-022-02347-3

**Published:** 2022-07-30

**Authors:** Milad Moghimi, Dana Jafarpour, Reihaneh Ferooz, Rafat Bagheri

**Affiliations:** 1 Tehran, Iran; 2 Faculty of Dental Medicine and Oral Health Sciences, McGill University, Montreal, Canada; 3 MSK Physiotherapy Pty Ltd, Melbourne, Australia; 4 Department of Dental Materials, Shiraz Dental School, Shiraz University of Medical Sciences, Shiraz, Iran

**Keywords:** Resin-based coating, Wear resistance, Glass ionomer restoratives

## Abstract

**Background:**

The effect of nanofilled resin-based coating on the wear resistance of glass ionomer cements (GICs) is still controversial. This study aims to compare the wear resistance of four encapsulated GICs including two conventional and two resin-modified, and to evaluate the effect of G-Coat Plus on the wear resistance of GICs.

**Methods:**

A total of 80 disk-shaped specimens were prepared from two CGICs (riva self cure (SDI) and Equia Forte Fil (GC) and two RM- GICs (Ketac Nano (3 M/ESPE) and Fuji II LC (GC). The specimens of each material were divided into two groups (n = 10) based on the surface protection: no coating (NC), and coating with G-Coat Plus (GCP). All specimens were then placed in distilled water for 24 h at 37 °C. The specimens were subjected to thermocycling for 120,000 cycles using a chewing simulator. Wear resistance was measured using a specific formula. Data was analyzed using Kruskal–Wallis test.

**Results:**

There was no significant difference in volume loss (mm^3^) between coated and uncoated groups for all materials (*P* > 0.05). Ketac Nano showed significantly lower volume loss (0.65 ± 0.12) compared to all other groups (*P* < 0.05) among uncoated specimen, and significantly lower than Fuji II LC (*P* = 0.035) and Equia Forte Fil (*P* = 0.040) among coated groups. However, no statically significant difference was observed between volume loss of coated Ketac Nano with that of riva self cure (*P* = 0.087).

**Conclusions:**

Coating with GCP did not affect the wear depth of GICs, and Ketac Nano showed significantly lower volume loss regardless of coating.

## Introduction

Over the past 45 years, glass ionomer cement (GIC) has evolved into diverse dental products used as direct restoratives, luting agents, liner and bases, pit and fissure sealants, endodontic sealers, atraumatic and minimum-invasive materials [1, 2]. GICs are the preferred choice for clinicians in the non-stress-bearing build-up, sandwich technique, root caries, and long-term provisional restorations [3]. They possess several advantages such as fluoride release, similar coefficient of thermal expansion as of the natural tooth and the facilitation in remineralization of caries-affected dentin [2, 3]. Biocompatibility and physicochemical bonding to enamel and dentine make them particularly favorable in restorative dentistry [4]. Despite these benefits, GICs are susceptible to early moisture sensitivity, which has been reported to be mitigated with the use of resin coatings [5]. In addition, their main disadvantage is low wear resistance in sites subjected to high occlusal force and lack of sufficient fracture toughness [6].

GIC entails specific properties which mark the material’s sensitivity to water. It consists of a basic aluminosilicate glass powder combined with an aqueous polymeric acid solution. To start the acid reaction, the polymeric acid needs water to release protons. Therefore, the setting reaction of GIC happens in the presence of water within 24 h after the material’s mixing, during which the GIC is sensitive to water exchanges [4]. If the premature GIC encounters moisture, it might lose its constituents, leading to surface wear and reduced translucency. On the other hand, if the reaction occurs in a dry condition, the GIC is likely to lose water, resulting in compromised adhesion, dimensional alterations, and internal crack formation, which diminishes the material’s strength [7, 8].

Therefore, coating materials such as varnishes, adhesive systems, petroleum jelly, and nanofilled self-adhesive light-cured protective coating (NPC) are introduced to protect the surface of GIC, to overcome its early sensitivity to moisture. Petroleum jelly is considered a good option due to its safety and biocompatibility [9] but can be easily washed away [10]. A long-lasting surface coating is desired to isolate the GIC from moisture during the entire setting period. Given this context, a new generation of coating (NPC) for GIC was developed (G-Coat Plus). G-Coat Plus (GCP) has shown to isolate GIC from saliva contamination during the complete maturation of the material, occlude surface cracks and porosity, and reinforce its strength [11–13]. Therefore, GCP is found to increase the wear resistance of GIC [14], without compromising the fluoride release [15] nor the caries-preventive effect [16].

The human oral environment is very dynamic, in which two fundamental wear mechanisms occur: (i) two-body abrasion, including when abrasive particles are bonded to abrasive instrument like dental bur, and (ii) three-body abrasion, including when abrasive particles are free between two surfaces, attrition, adhesion, fatigue and erosion or any combination of these interactions [17, 18]. The in vitro wear is determined using two methods. In the first method, the type of movement is used to measure the wear rates which include: adhesive wear when occlusal cusps contact a GIC, abrasive wear that can be categorized as two- and three-body wear, and fatigue due to cyclic loading that lead to loss of materials (reciprocating, rolling, impact oscillation and flow). The second method of wear rate measurement is based on the machines which stimulate chewing and the wear processes associated with it. The Oregon Health Science University Oral wear simulator (OHSU) and the BIOMAT chewing simulator are two such examples [18–20].

Although the effect of GCP on the mechanical and physical properties of GICs and resin composites has been evaluated previously [11–13, 21–23], its effect on the wear resistance of GICs is still controversial. Therefore, the present study aims to compare the wear resistance of four encapsulated GICs; two conventional (CGIC) and two resin-modified (RM-GICs), and to evaluate the effect of a nanofilled resin-based coating on the wear resistance of those GICs. The null hypothesis was that there would be no difference among the materials, and that the surface coating has no effect on wear resistance of GICs.

## Materials and methods

The present study was approved by the ethics committee (#9761) at Shiraz University of Medical Sciences, Shira, Iran. The materials used in this study are shown in Table 1. A total of 80 disk-shaped specimens of 10 mm diameter and 2 mm thickness were prepared (20 for each material). A polyethelyn mould was placed on the top of a Mylar strip on a glass plate and filled with the material according to the manufacturer's instructions. To remove excess material, a second piece of Mylar strip was placed on the material in the mould and pressed by another glass plate under hand pressure. The resin-modified specimens were irradiated for the recommended exposure time through Mylar strip using LED curing unit (Radii plus LED; SDI, Victoria, Australia) with a wavelength of 440–480 mm, emitting light intensity of 1500 mW/cm^2^. The specimens for each material were randomly divided into two groups: coated and uncoated (n = 10). For the coated groups, the specimens were immediately coated with GCP on one side where the force would be applied, and light cured according to the manufacturer’s instructions. The specimens were marked and placed in distilled water at 37 °C for 24 h prior to being tested.

**Table 1:** Material description and manufacturer's details

Materials	Manufacturer	Composition	LOT number
Ketac™ Nano	3 M ESPE, California, USA	Fluoro-aluminosilicate glass/ Polyacrylic acid/Tartaric acid	NA07509
Riva self cure	SDI, Victoria, Australia	Fluoro-aluminosilicate glass/ Polyacrylic acid/Tartaric acid	C1712053F
Fuji II LC	GC Corporation, Tokyo, Japan	Aluminium-fluoro-silicate glass/Poly-HEMA	1,803,061
Equia Forte Fil	GC Corporation, Tokyo, Japan	Fluoro-alumino-silicate glass/Polybasic carboxylic acid/Polyacrylic acid/Distilled water	1,803,131
G-Coat Plus	GC Corporation, Tokyo, Japan	Urethane methacrylate/Methyl methacrylate/Camphorquinone/Silicon dioxide/Phosphoric ester monomer	1,807,041

After 24 h, the specimens and antagonists were mounted in the mastication simulator (CS4, SD Mechatronik, Feldkirchen, Germany). Antagonist were prefabricated stainless-steel beak. This creates a two-body abrasion on the specimens’ surface and simulates the antagonist teeth in mouth and make wear effect on the outer side of them during a particular time. Two Kilogram weights were used in each chamber and the sliding movement was set to 0.7 mm. The frequency of the antagonist movement was 1.5 Hz, and each mastication cycle was repeated 120,000 times.

**Fig. 1:**
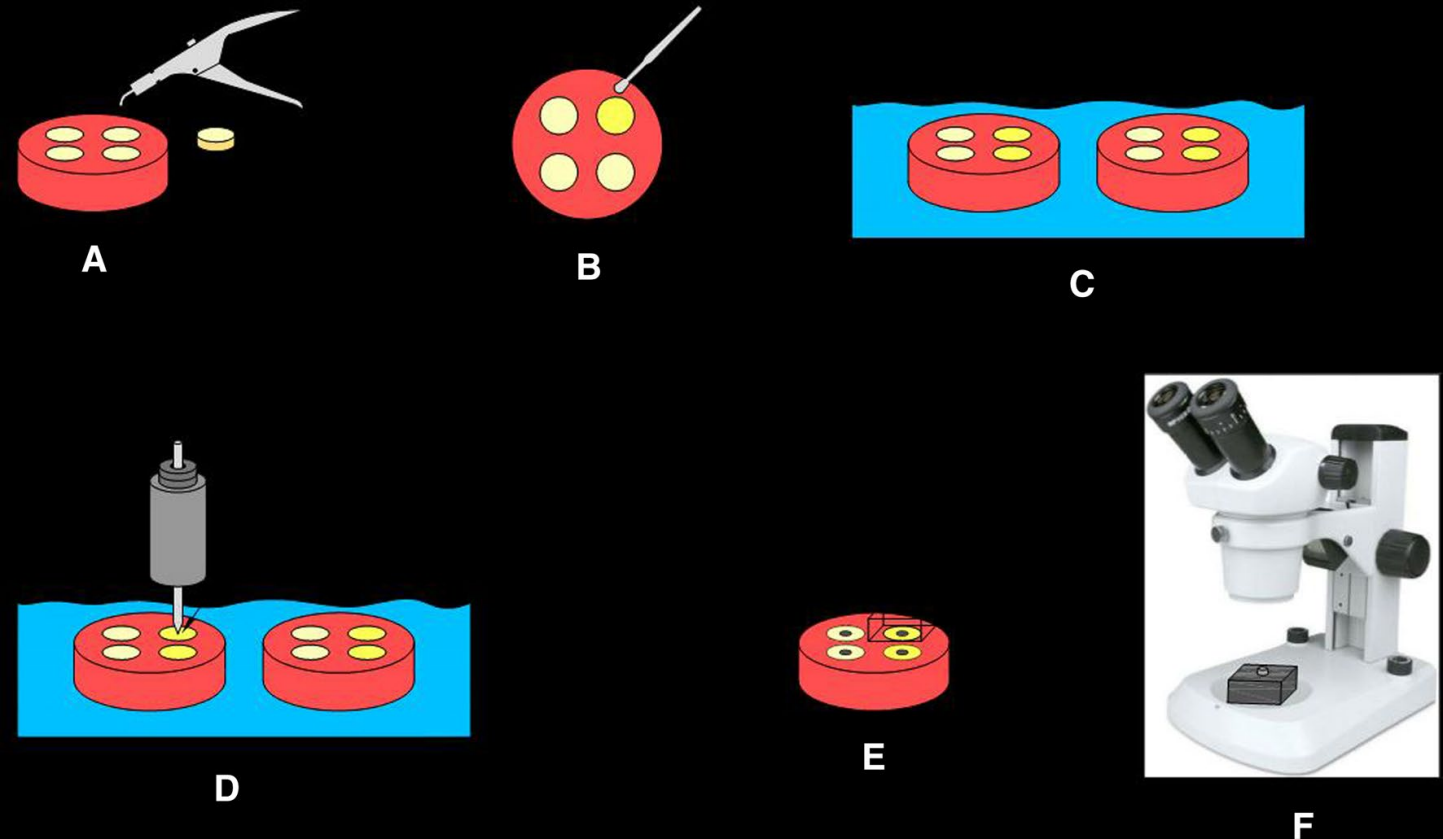
Schematic draw of the specimens’ treatment and wear measurement. **A**: Specimen preparation; **B**: Application of resin coating; **C**: Immersion in distilled water; **D**: Chewing simulation; **E**: Impression taking of the loaded surface; **F**: Evaluation under stereomicroscope

During the in vitro mastication process, water at 37 °C was used to simulate the oral environment and to remove worn particles from the materials’ surface. After 120,000 loading cycles, an impression of the loaded surface for each specimen was taken using an additional silicon impression material (Spidex, Coltene, Altstatten, Switzerland). Schematic draw of the specimens’ treatment and wear measurement is shown in Fig. 1.

**Fig. 2:**
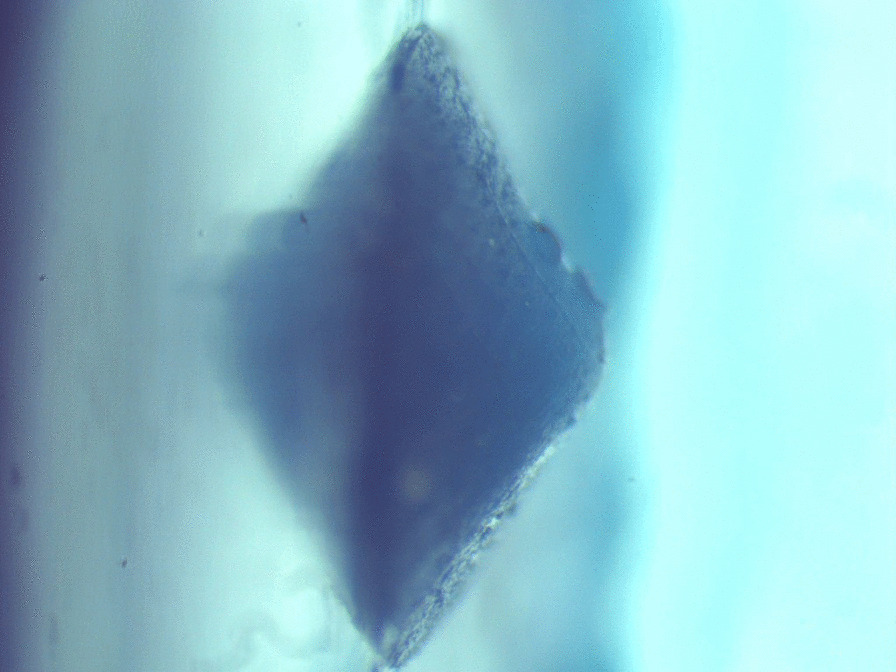
A replica of specimen surface after chewing simulation was done. This photo was taken under the stereomicroscope

**Fig. 3:**
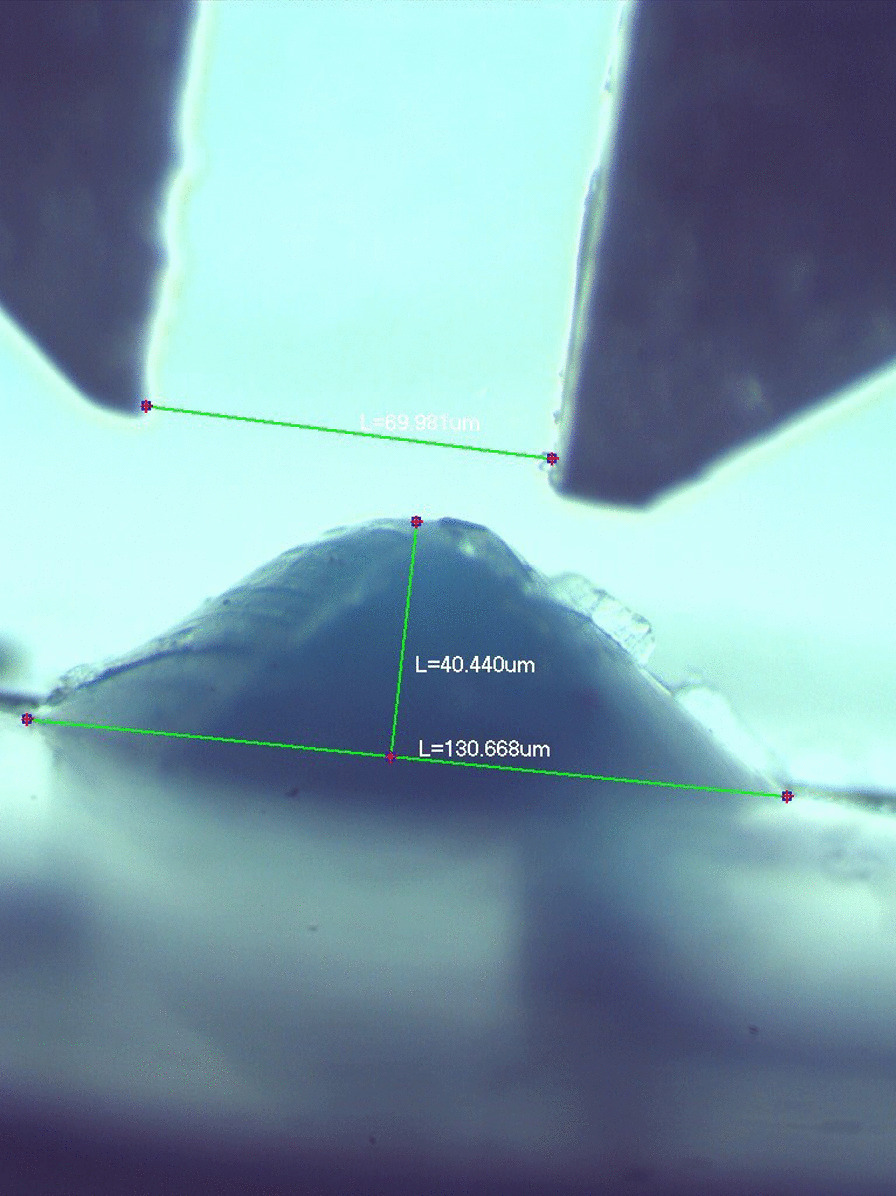
The vertical and volume loss were calculated under the stereomicroscope

Vertical substance loss (Fig. 2) of the specimen was measured by observing the replica under a stereomicroscope (BestScope, BS-3060C, Beijing, China). The deepest point of the profile represented the vertical substance loss. The wear area was analyzed with the stereomicroscope which was connected to a digital camera (BestScope, BS-3060C, Beijing, China) (Fig. 3). The radius of wear area was directly measured using the software Scope Image, version 9.0 (BP Integrated Technologies, Calamba city, 4027 Philippines) at a magnification of 25 × . As the wear area resembled a spherical segment, the volume loss was mathematically calculated in sufficient approximation using the following formula for a spherical segment:

$${\text{V}} =\uppi /6{\text{h }}(3\uprho ^{2} + {\text{ h}}^{2} ).$$

where V = volume loss in mm^3^, h = vertical loss in mm, ρ = radius of wear in mm.

### Data analysis

The data were collected using a statistical software (SPSS Statistics for Windows, Version 22.0; IBM, Armonk, NY, USA). Median and interquartile range were used to report the data. The Kruskal–Wallis test was used to analyze the data. The Pearson correlation coefficient was calculated to determine if there was a relationship between the vertical loss and volume loss values. A P value less than 0.05 was considered statistically significant.

## Results

The median and the interquartile range of vertical and volume loss of the materials tested in this study are presented in Table 2.

**Table 2:** Median ± interquartile range of vertical loss (mm) and volume loss (mm^3^)

Material		Uncoated	Coated	*P*-Value
Fuji II LC	Vertical loss	0.53 $\pm$ 0.47	0.69 $\pm$ 1.74	–
	Volume loss	1.48 $\pm$ 0.48^**A**^	2.12 $\pm$ 1.75^**A**^	0.966
Ketac nano	Vertical loss	0.50 $\pm$ 0.12	0.55 $\pm$ 0.17	–
	Volume loss	0.65 $\pm$ 0.12^**B**^	0.76 $\pm$ 0.17^**B**^	0.368
Equia forte fil	Vertical loss	0.37 $\pm$ 0.21	0.65 $\pm$ 5.49	–
	Volume loss	0.95 $\pm$ 0.21^**A**^	1.70 $\pm$ 5.49^**A**^	0.333
Riva self cure	Vertical loss	0.76 $\pm$ 2.15	0.60 $\pm$ 1.79	-
	Volume loss	1.52 $\pm$ 2.15^**A**^	1.47 $\pm$ 1.79^**AB**^	0.784

Different upper-case letter shows significant difference between materials in each coating (in a column)

The results of vertical loss in Table 2 exhibit that among uncoated groups, Equia Forte Fil had the lowest value (0.37 ± 0.21), followed by Ketac Nano, Fuji II LC and riva self cure. In the coated groups, Ketak Nano (0.55 ± 0.17) showed the lowest vertical loss and Fuji II LC the highest (0.69 $\pm \hspace{0.17em}$1.74).

As shown in Table 2, there was no significant difference in volume loss (mm^3^) between coated and uncoated groups in any of the materials (*P* > 0.05). Ketac Nano showed significantly lower volume loss than all other materials in the uncoated groups (0.65 ± 0.12) (*P* < 0.05).

Among coated groups, the volume loss value for Ketac Nano was significantly lower than that of Fuji II LC (*P* = 0.035) and Equia Forte Fil (*P* = 0.040) but no statistically significant difference was observed with that of riva self cure (*P* = 0.087).

As shown in Table 3, there was a significant positive correlation between volume and vertical loss in all tested materials except for the uncoated Ketak Nano in which no significant correlation was found between the two values (*P* = 0.120).

**Table 3:** Pearson correlation values between vertical and volume loss in each material

Material		Uncoated	Coated
Fuji II LC	r^*^	0.97	0.99
	t^**^	8.31	12.66
	p^ϯ^	0.00	0.00
Equia forte fil	r	0.97	0.79
	t	8.67	2.54
	p	0.00	0.03
Ketac nano	r	0.56	0.74
	t	1.37	2.20
	p	0.12	0.05
Riva self cure	r	0.94	0.80
	t	5.72	2.69
	p	0.00	0.03

* $$r = \frac{{\left[ {\frac{1}{N - 1}} \right]\sum {\left( {x - \overline{x}} \right)\left( {y - \overline{y}} \right)} }}{{s_{x} s_{y} }}$$ ** $$t = \frac{{r\sqrt {N - 2} }}{{\sqrt {1 - r^{2} } }}$$ ^**ϯ**^ P value

## Discussion

The present study was designed to compare the wear resistance of encapsulated conventional and RM-GICs, and to determine if surface coating of GICs by nanofilled resin-based agent has a protective effect against vertical and volume loss of the tested materials. The null hypothesis was partly rejected as the findings revealed no statistically significant difference between the wear resistance of the coated and uncoated GICs. However, there was significant difference between materials of both groups.

Wear resistance is considered as one of the most imperative properties for all dental materials in the oral environment. Wear resistance is the capability of the restoration to endure the grinding force of the opposite tooth and food concurrently, whilst upholding its function. Regardless of some favorable properties, GICs have been proven inappropriate for stress-bearing sites because of their poor wear resistance [24, 25]. Therefore, the application of GCP has been recommended to improve mechanical and physical properties of GICs. In a previous study, Bagheri et al. [26] showed that application of GCP has a significant increase on the shear punch strength of Fuji II LC and Fuji IX after 48 h and 8 weeks immersion in distilled water. In another study, they also showed that surface coating significantly increased flexural strength of the most tested GIC materials [12]. Jafarpour et al. [27] found that coating the GIC restorations decreased water sorption and solubility of almost all tested materials [riva self and light cure (SDI), Fuji II LC and Equia forte Fil (GC)] and reduced their susceptibility to staining. It is speculated that the protective effects of GCP allows complete maturation of the GIC’ reaction, with delayed moisture contamination, preventing water sorption solubility at the same time as creating a stronger material. Therefore, to examine the effect of coating on wear resistance of GICs, GCP was implemented in the present study. The chewing stimulator was used in this research to simulate mechanical loading and thus increase the clinical relevance of the study. This device is a two-body wear test machine, with its main mechanism in the present study: abrasion in combination with surface fatigue [28]. The specimens were loaded in a biaxial chewing simulator, 120,000 chewing cycles were performed with 700 thermal cycles, which corresponds to a clinical service time of about 6 months.

The application of nanofilled coating in the present study showed no positive effect on the wear resistance of the tested materials. This could be explained by the fact that the coating keeps the material from wear by tearing itself. However, after a certain number of cycles, its protective effect will be lost as the coating will be completely worn out from the surface [29]. Our results could be justified by the findings of Bagheri et al. [26] which found the application of GCP lead to a significant decrease in the hardness of the GIC restorations. This may be due to the resin-enriched top layer, which is a much weaker phase than the bulk of the cured material and its application cannot reduce the surface wear of GICs. Previous studies have been controversial regarding the effectiveness of coating on wear resistance of glass ionomer materials. While Bonifacio et al. [30], showed significant improvement in the wear resistance of Fuji IX GP Extra when G-Coat Plus was applied, Kielbassa et al.’s [31] findings on Equia coating supports our outcome by showing a lack of effective long-term protection against abrasive wear for Equia coat. Our finding is also in agreement with that of Rye et al. [32] who reported no statistically significant differences in the wear resistance between coated and uncoated GICs. Moreover, Bertrand et al. [33] revealed that the application of resin coating caused decreased microhardness of the composite resin's surface. In another recent study on the wear resistance of resin coated GICs, Brkanović and colleagues [34] reported that the resin coating does not significantly increase wear resistance. Contrary to our findings, a previous clinical study [14] proposed that the surface protection with G-coat plus had a protective effect on the clinical wear of GIC approximal restorations in primary molars after 3 years. However, the study did not use an uncoated control group in their methodology, and thereby, their conclusion may not be as reliable. In other work by Ryu et al. [32] on Equia coat, the authors reported an increase in wear resistance followed by surface protection. The difference in the tested coating as well as fewer chewing cycles implemented in their study may explain the contradictory finding. According to Bonifacio et al. [30], a micromechanical interlocking was reported between the GCP and the Fuji IX GP Extra under SEM. These findings might suggest that GCP is advantageous in decreasing the early wear when used with Fuji IX GP Extra [30].

The results of our study revealed a significantly higher wear resistance for one of the RMGICs (Ketac Nano) tested compared to the conventional GICs. This result was expected as the addition of the resin component to the CIC was aimed to improve the wear resistance and physical strength of the cement [35]. Previously, studies have substantiated superior physical properties and high initial strength for RMGICs due to their resin monomer. Croll and Nicholson [35] demonstrated improved fracture toughness, fracture resistance, and wear resistance for RMGICs. Furthermore, the suitable properties which were fluoride ion hydrodynamics, biocompatibility, favorable thermal expansion and contraction properties, and physiochemical bonding to tooth structure, remain devoid of any degradation. They also proposed that the best mechanical features were reached when the least amount of liquid was used to wet the powder. Daniela S. Rodrigues et al*.* [36] also verified the higher wear resistance of RMGICs compared to conventional ones. However, Lohbauer [37] stated that RMGICs are more disposed to abrasive wear because of a fragile filler matrix coupling and because the added resin monomer and supplementary photo polymerization could not pass the dehydration constraint. This dictates the need for resin coating for at least 1 h immediately after restoration. In the present research, Ketac Nano, which is a newly introduced RMGIC, showed the least volume loss values among the tested materials regardless of the coating application. According to the manufacturer’s claims, Ketac Nano is the first nano-ionomer developed in Dentistry. In addition to the classic GIC fluor aluminosilicate glass, this nano-ionomer comprises silane-treated silica nano-fillers and clusters of single unit nano-sized silica/zirconia, leading to greatly packed filler composition [33]. The findings of the present study substantiate the manufacturer's claims regarding the material’s improved wear resistance compared to that of Fuji II LC, a traditional RMGIC.

As the findings revealed, there was a positive correlation between the volume and vertical loss in all tested materials. Indicating that the alterations observed between the volume loss values of different materials can be attributed to the corresponding vertical losses. The diameter of the abraded geometry was not different between groups following chewing simulation.

Previous work has shown that artificial saliva and water are mostly comparable as storage media in regards to water sorption [38]. As shown in a previous study [39], distilled water can have similar effects to artificial saliva when glass ionomer cements are coated; therefore, it was used as a storage medium in the present study. We used distilled water so that our findings would be comparable to the findings of previous studies which used water as the immersion media. For instance, Bagheri et al. [26] showed that the application of GCP has a significant increase on the shear punch strength of Fuji II LC and Fuji IX after 48 h and 8 weeks immersion in distilled water.

One of the limitations of this study was that wear resistance was measured in approximation considering the potential errors using an impression material. Different studies use varying clinical wear measurements making the method unreproducible due to interpersonal variations, making analysis even harder. Although chewing simulator was performed to imitate chewing in the present study, the procedure was still conducted in the laboratory setting where the complete simulation of oral environment was not possible. Thus, further clinical studies are required on this topic. We recommend that randomized clinical trials be carried out on the physico-mechanical properties of glass ionomer restorative materials coated in vivo. Additionally, the specimens were stored in water for only 24 h, therefore the long-term effect of the surface coating should be further evaluated, as solubility of resin-based materials in water occur mostly due to the leaching of free residual monomers [40]. We suggest that the effect of aging be taken into account in future research.

## Conclusion

Based on the results of this study, the following conclusions may be drawn: application of surface protection did not have a major influence on the wear resistance of the conventional and resin modified GICs; thus, wear resistance appeared to be independent of surface coating. However, the wear resistance relied on the type of materials. Amongst the coated materials studied, Ketac Nano showed the least vertical and volume loss followed by riva self cure, Equia Forte Fil, and Fuji II LC.

### Clinical significance

Surface coating may not improve the wear resistance of glass ionomer restorative materials.

## Data Availability

The datasets used and/or analysed during the current study are available from the corresponding author on reasonable request.
